# Development and Validation of Indicators for Population Injury Surveillance in Hong Kong: Development and Usability Study

**DOI:** 10.2196/36861

**Published:** 2022-08-18

**Authors:** Keith T S Tung, Rosa S Wong, Frederick K Ho, Ko Ling Chan, Wilfred H S Wong, Hugo Leung, Ming Leung, Gilberto K K Leung, Chun Bong Chow, Patrick Ip

**Affiliations:** 1 Department of Paediatrics and Adolescent Medicine The University of Hong Kong Hong Kong Hong Kong; 2 Institute of Health and Wellbeing University of Glasgow Glasgow United Kingdom; 3 Department of Applied Social Sciences The Hong Kong Polytechnic University Hong Kong Hong Kong; 4 Accident and Emergency Department Princess Margaret Hospital Hong Kong Hong Kong; 5 Department of Surgery The University of Hong Kong Hong Kong Hong Kong

**Keywords:** injury, indicators, modified Delphi research design, surveillance

## Abstract

**Background:**

Injury is an increasingly pressing global health issue. An effective surveillance system is required to monitor the trends and burden of injuries.

**Objective:**

This study aimed to identify a set of valid and context-specific injury indicators to facilitate the establishment of an injury surveillance program in Hong Kong.

**Methods:**

This development of indicators adopted a multiphased modified Delphi research design. A literature search was conducted on academic databases using injury-related search terms in various combinations. A list of potential indicators was sent to a panel of experts from various backgrounds to rate the validity and context-specificity of these indicators. Local hospital data on the selected core indicators were used to examine their applicability in the context of Hong Kong.

**Results:**

We reviewed 142 articles and identified 55 indicators, which were classified into 4 domains. On the basis of the ratings by the expert panel, 13 indicators were selected as core indicators because of their good validity and high relevance to the local context. Among these indicators, 10 were from the construct of health care service use, and 3 were from the construct of postdischarge outcomes. Regression analyses of local hospitalization data showed that the Hong Kong Safe Community certification status had no association with 5 core indicators (admission to intensive care unit, mortality rate, length of intensive care unit stay, need for a rehabilitation facility, and long-term behavioral and emotional outcomes), negative associations with 4 core indicators (operative intervention, infection rate, length of hospitalization, and disability-adjusted life years), and positive associations with the remaining 4 core indicators (attendance to accident and emergency department, discharge rate, suicide rate, and hospitalization rate after attending the accident and emergency department). These results confirmed the validity of the selected core indicators for the quantification of injury burden and evaluation of injury-related services, although some indicators may better measure the consequences of severe injuries.

**Conclusions:**

This study developed a set of injury outcome indicators that would be useful for monitoring injury trends and burdens in Hong Kong.

## Introduction

Injuries, including both unintentional injuries and violence, are serious public health threats that accounts for approximately 10% of the world’s fatalities [1]. Similar to other countries, injuries are a significant public health problem in Hong Kong. Injuries have consistently been among the top 5 causes of mortality since 2001 and accounted for approximately 1850 registered deaths in 2019 [2]. In addition, at least 6.2% of the population has experienced functional impairment resulting from at least one episode of unintentional injury [3]. Injuries were also found to be the leading cause of death in the age group of 1 to 14 years over the past decades [2].

Thus, it is important to develop robust strategies to monitor and prevent injuries. However, quantification of the injury burden is a challenging process because of considerable variations in injury mechanisms, duration, and outcomes [4,5]. This process should be guided by a set of measurable injury indicators [6,7]. Broadly, indicators are defined as derivatives of primary data that provide information and describe the state of a phenomenon to a degree of significance beyond raw measurements [8]. Establishing a set of injury indicators would provide a standardized tool to estimate the local injury burden and increase the validity and comparability of these estimates between populations [9].

As the occurrence of injuries partly depends on environmental and social factors [4,5], injury patterns could vary substantially, even within the same population and across regions [10]. The use of local context-specific indicators is recommended to standardize the definitions of injuries and increase the reliability and representativeness of the results in reflecting the injury situation at the population level [11]. Country-specific sets of indicators have been established in Western countries, including Canada, the Netherlands, and the United Kingdom [6,12,13]. However, there has been no comprehensive review of the injury indicators specific to Hong Kong. Hong Kong has 18 districts, each with unique demographic, environmental, and socioeconomic characteristics. It is also known for extremely high–living density multistory apartments, and thus, its injury patterns could be different from those in other regions. Therefore, this study aimed to develop a valid set of Hong Kong–specific injury indicators through a multi-phased modified Delphi research design. The resulting insights would be beneficial for the planning, implementation, and evaluation of injury surveillance and prevention programs [14]. The surveillance system, in turn, can provide information for the early identification of warning signs of injuries and timely intervention for individuals who may be at risk of a physical or psychological injury, ultimately reducing health care use and spending at both individual and societal levels [6,7].

## Methods

### Ethics Approval

The study protocol was approved by the Institutional Review Board of the Hospital Authority, Hong Kong West Cluster (reference UW 15-549). Informed consent was not required from participants as all data provided by the Clinical Data Analysis and Reporting System (CDARS) were deidentified.

### Study Design

#### Overview

The development of injury indicators adopted a multiphase modified Delphi research design, as described in previous studies [6,7]. Individuals can first express their opinions impersonally, followed by a whole-group discussion to reach a consensus [15]. The Delphi process emphasizes collective expert opinions rather than precise analytical techniques [16], which makes it particularly suitable for studying population-level research questions or problems. Although the original Delphi process includes a series of iterative steps to collect aggregated expert opinions through multiple rounds of questionnaires, the modified version adopted in our study initiated the discussion with a list of carefully preselected items to facilitate the process of reaching consensus. Following the modified Delphi process protocol, 5 phases were involved: (1) searching and reviewing relevant studies from academic databases, (2) extracting potential indicators from identified studies, (3) achieving a consensus opinion among experts on locally relevant indicators, (4) specification of the selected indicators, and (5) applicability testing of the selected indicators using local health data.

#### Phase 1: Searching and Reviewing Relevant Studies

A scoping review was conducted to identify previously adopted valid injury indicators by summarizing the evidence from the included studies that met the prespecified inclusion criteria [17]. This is an evidence-based method to create a rich database as groundwork for further research or review and has been used in previous studies to investigate different research questions and topics [18,19]. Specifically, a literature search was conducted to identify existing outcome indicators for all types of injuries that require medical attention, both intentional and unintentional, from academic databases, including ProQuest, Web of Science, PubMed, Ovid, PsycINFO, and Google Scholar. Guided by the International Classification of Diseases-9), which focuses on the consequences of injuries, the following search terms were combined in various ways to perform the search: *burns*, *poisoning*, *dislocations*, *drowning*, *road traffic accident*, *facial trauma*, *head trauma*, *internal injury of thorax*, *abdomen and pelvis*, *fracture*, *internal injury*, *injury to nerves*, *injury to spinal cord*, *open wounds*, *falls*, *blunt injury*, *suicide*, *self-harm*, *self-inflicted injury*, *injury*, *outcome*, *consequence*, *intentional*, *unintentional*, *psychological*, *mental health*, *mental disorders*, *Abbreviated Injury Scale*, *Glasgow Coma Score*, *Injury Severity Scale*, and *disability*.

The articles included in this review met the following inclusion criteria: (1) having an injury case definition, (2) including at least one indicator of outcome after injury, (3) providing possible data source or sources for the indicator or indicators, (4) being published in an academic peer-reviewed journal, and (5) written in English.

#### Phase 2: Extracting Potential Indicators From Selected Studies

The aim of this phase was to extract relevant information from the selected studies for subsequent expert reviews. The extracted information included the study year, type of injury, severity of the injury, study population, outcome indicators, and type of study. All the data were recorded and compiled in a spreadsheet for further analysis. Data extraction was conducted by trained research assistants under the supervision of experienced researchers from various fields, including social sciences, medicine, statistics, and biology. The purpose of including a cross-disciplinary and diverse research team was to ensure the accuracy of extracted information. On the basis of their characteristics and measurement purpose, the indicators identified from the literature were grouped into 4 constructs: health care service use, functional and psychological outcomes, biological and physiological outcomes, and postdischarge outcomes.

#### Phase 3: Achieving Consensus Opinion Among Experts on Locally Relevant Indicators

The indicators identified in the literature were considered suitable for potential use in Hong Kong. The list of potential indicators identified by the research team was sent to a panel of 18 experts in the field of injury for evaluation. Experts from different sectors, including the government, academia, health care, and the community, are well recognized for their work and contributions to injury measures, data management, and community safety and prevention programs. A 22-item checklist adopted from Pike et al [6] and the Child Health Indicators of Life and Development project [20] was used to evaluate the validity, consistency, local relevance, and sensitivity of the potential indicators. The evaluation process involves 2 steps. The first step was a web-based survey inviting experts to view and rate the indicators according to checklist criteria. The second step was face-to-face discussion to resolve disagreements among experts [21]. For each indicator, the response of *YES* denotes satisfactory fulfillment of the specific criterion, whereas the response of *NO* denotes a failure to fulfill that criterion. Indicators receiving *YES* for ≥11 prespecified criteria from more than half of the panel members were deemed as the core locally relevant injury indicators. In addition, panel members were asked to propose other suitable indicators to be included in the list. The newly proposed indicators are then circulated among the panel members for a second round of review.

#### Phase 4: Specification of Selected Indicators

In this phase, core indicators were clearly defined and specified by the research team according to the specification format adopted from previously published reports on injury indicators [22-24]. Each indicator has its own specification items, including the definition of the indicator and relevant terms, justification for its inclusion, operational definition of a case, method and tools for calculation, data sources and availability, units of measurement, and limitations.

#### Phase 5: Applicability Testing of Selected Indicators Using Local Health Data

The applicability of these core indicators was evaluated through a series of regression analyses using local health data. Regression analyses were conducted to test the associations between core indicators and certification of the Hong Kong Safe Community in different districts in Hong Kong. The Safe Community model aims to promote safety through the prevention of accidents and injuries through cross-sectorial collaboration in the community [25]. To be certified as a Hong Kong Safe Community, the district must fulfill the following six criteria: (1) establishment of community safety policy, (2) establishment of Safe Community organization, (3) identification of community safety needs, (4) implementation of community safety programs, (5) evaluation of community safety programs, and (6) sharing of safety community experience [25]. Previous studies have demonstrated a reduction in injury incidence after the implementation of the Safe Community model [26,27]. To test the applicability of the core indicators for the quantification of injury burden, all data concerning accident and emergency department (AED) attendance (based on a trauma flag entered by nurses) and hospitalization (based on International Classification of Diseases-9 codes) attributable to injuries during the period from January 1, 2001, to December 31, 2016, were extracted from the CDARS. The CDARS is a territory-wide electronic health record database managed by the Hospital Authority, which is the official governing body of all public hospitals in Hong Kong. All AED attendance and inpatient records of the Hong Kong local public hospitals are housed in the CDARS. A difference-in-differences comparison was adopted in our multivariable regression models to examine the effects of the Hong Kong Safe Community model. The time-varying injury indicator variables were the outcomes. The rate indicators were modeled using a negative binomial model with the log-transformed population during that period to be the offsets. Continuous indicators were modeled using linear regression models. The primary independent variable of interest was a time-varying binary variable with *0* and *1* indicating pre and post–Safe Community implementation. The overall year trend was adjusted for in the model as a continuous variable. This approach can help minimize the temporal influences on injury incidence in Hong Kong.

## Results

### Phase 1 to Phase 2

**Figure 1:**
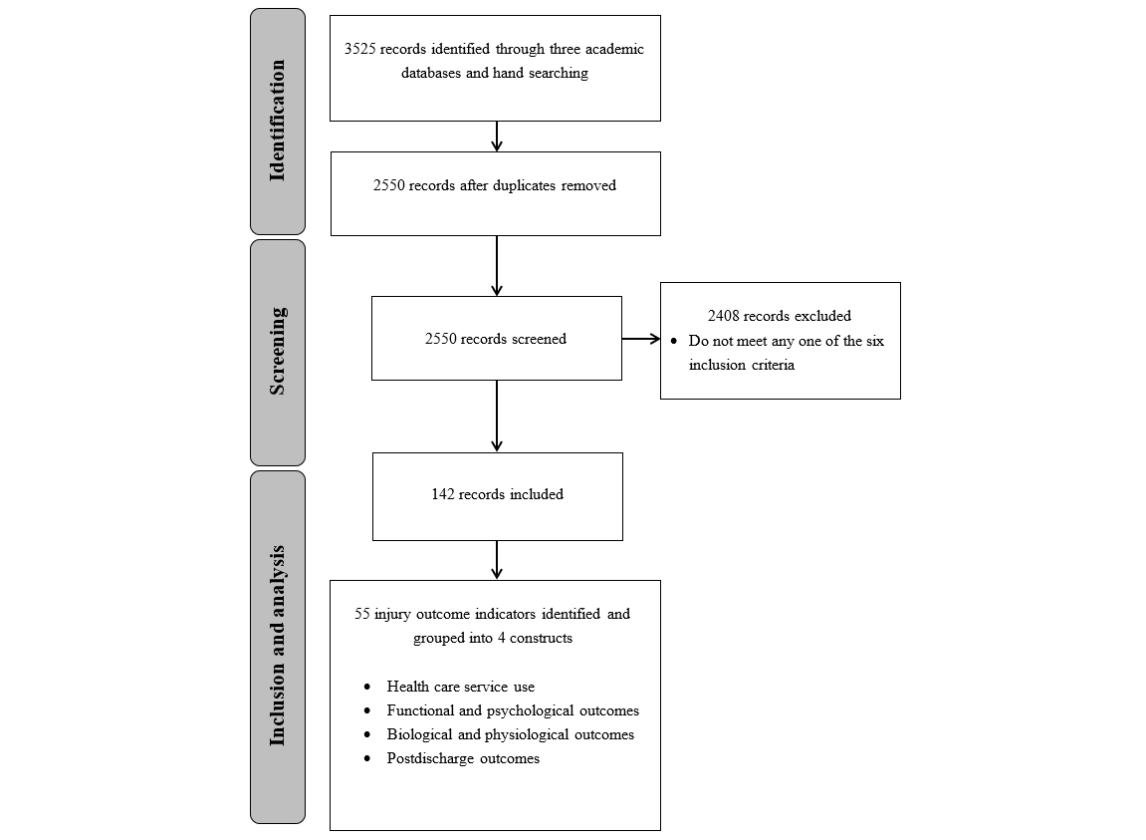
Flow diagram of the scoping review.

The initial literature search in phase 1 identified 3525 records. Among these 3525 records, 142 (4.03%) met our prespecified inclusion criteria and were included in the review process (Figure 1). From the 142 reviewed articles, we identified 55 injury outcome indicators and categorized them into 4 constructs based on their characteristics: functional and psychological outcomes (19/55, 35% indicators), health care service use (17/55, 31% indicators), postdischarge outcomes (10/55, 18% indicators), and biological and physiological outcomes (9/55, 16% indicators). Owing to the high heterogeneity of information within the construct of functional and psychological outcomes, indicators in this construct were further divided into 5 subcategories: integrated outcomes (6/19, 32% indicators), lower limbs (5/19, 26% indicators), cognitive (3/19, 16% indicators), psychiatric (3/19, 16% indicators), and upper limbs (2/19, 11% indicators). In contrast, as most indicators in the construct of biological and physiological outcomes were injury specific, they were further regrouped into 9 broad categories based on their similarities to expedite the expert review process. Table 1 displays all indicators identified from the literature by the research team.

**Table 1:** Potential injury outcome indicators under the 4 constructs.

Indicator				Definition
**Health care service use indicators**				
	Operative intervention [28-44]			The percentage of cases that requires operative intervention within a particular year
	Infection rates [28,45-53]			The percentage of cases that have any type of infection within a particular year
	Admission rate to ICU^a^ [30,32,33,35,38,44,54-67]			The percentage of cases admitted to the ICU within a particular year
	Immediate mortality rate [32,33,35-41,44-46,50,54-60,62,64-66,68-105]			The cause-specific death rate per 100,000 population within a particular year
	Length of ICU stay [40,44,61,62,64,67,90,98,103,106]			The average number of days spent in the ICU within a particular year
	Length of hospitalization [30,31,33,34,36,40-42,44-48,50,52,54-56,61-64,68,71,72,75,76,78,87,90,98,99,103-117]			The average number of days spent in hospital for a particular year
	Admission rate to AED^b^ [43,66,69,100-102,117-121]			The cause-specific admission rate to the AED per 100,000 population for a particular year
	Time for wound healing [50,109]			Average amount of time required for the healing of injury-induced open wounds within 1 particular year
	Need for rehabilitation facility [30]			Total number of cases that need to be referred to an inpatient rehabilitation facility within a particular year
	Hospitalization rate after leaving AED [30,35,38,53-55,58,60,61,66,69,77,83,100-102,105,107,108,111,117-120,122,123]			The percentage of cases that require inpatient hospital admissions after leaving AED per 100,000 population within a particular year
	Intubation duration [30,52,62,64,80,90,103]			The average number of days that the cases require intubation, a process of inserting through the mouth into the airway to assist with their breathing, within a particular year
	Need for secondary procedures [31-34,48,115]			The percentage of cases that need secondary surgical procedures within a particular year
	Mean duration of operation [37]			The average amount of time needed for the operative intervention conducted within a particular year
	Presence of complications [32,33,36,37,46,49,51,58,63,78,86,92,106,109,113-115,124,125]			The percentage of cases that have injury-induced complications within a particular year
	Morbidity [46,60,83-85,88,95]			The frequency of having any injury-induced disease or medical condition within a particular year
	Discharge rate [70,80,97-99,105,117,120,126]			The percentage of cases discharged from the hospital within a particular year
	Need for nursing facility [89]			Total number of cases discharged to a nursing facility within a particular year
**Functional and psychological outcome indicators**				
	**Cognitive outcomes**			
		Gray Oral Reading Test [127]	An efficient and objective measure of growth in oral reading fluency and comprehension and an aid in the diagnosis of oral reading difficulties	
		Cognitive Performance Scale [89,106]	A scale used for accessing patients’ loss in everyday cognitive performance from independent to full dependency based on 5 domains: daily decision-making, eating self-performance, ability to make self-understood, short-term memory, and being comatose	
		Stanford-Binet Intelligence Scale [127]	A cognitive ability and intelligence test used to diagnose developmental or intellectual deficiencies based on 5 factors: knowledge, quantitative reasoning, visual-spatial processing, working memory, and fluid reasoning	
	**Psychiatric outcomes**			
		Structured Clinical Interview for DSM-5^c^ or other related mental health scales [112,117,121,128-131]	A semistructured interview guide for making DSM-5 diagnoses or other mental health scales not stated	
		General Health Questionnaire [132]	A screening device for identifying minor psychiatric disorders in the general population and within the community or nonpsychiatric clinical settings such as primary care or general medical outpatients	
		Health Status Questionnaire [29,109,133-135]	A scale for measuring health-related quality of life, including physical functioning, role limitations owing to physical problems, bodily pain, general health perceptions, vitality, social functioning, role limitations because of emotional problems, and mental health	
	**Upper limb outcomes**			
		American Shoulder and Elbow Surgeons Evaluation [136]	A standardized method for assessing patient-rated shoulder pain and function or disability	
		Disabilities of the Arm, Shoulder, and Hand Questionnaire [137]	A region-specific outcome instrument developed as a measure of physical function and symptoms in patients with any or several musculoskeletal disorders of the upper limb	
	**Lower limb outcomes**			
		Walking Index for Spinal Cord Injury [138]	A research tool in clinical trials to measure improvements in walking in person with acute and chronic spinal cord injury	
		Lysholm score and Tegner Activity scale [139]	A scale used for measuring patients’ everyday activity limitations and participation restrictions before and after arthroscopic knee surgery	
		American Knee Society score [140]	A scale used with patients with osteoarthritis or who have undergone total knee arthroplasty for measuring patients’ functionality and their knee clinically through physical examination	
		Foot and Ankle Outcome score [28,141]	A scale used with patients with lateral ankle instability, Achilles tendinosis, and plantar fasciitis for assessing foot and ankle pain, symptoms, function in daily living, sport and recreation, and foot- and ankle-related quality of life	
		American Spinal Injury Association Impairment scale [28]	A scale used by the rehabilitation team to assess the sensory and motor levels that were affected by the spinal cord injury	
	**Integrated outcomes**			
		Neurological impairment [59,60,83,142,143]	A broad group of disorders in which the central nervous system does not function properly and leads to some form of physical or mental problems	
		Musculoskeletal Function Assessment [29,124,137]	An instrument used with a range of patients with musculoskeletal disorders for measuring their health status, including lower and upper mobility, activity level and satisfaction, social support, pain, emotional status, and quality of life, in clinical practice	
		Functional Independence Measure [98,127,135,144]	A basic indicator of patient disability and the amount of assistance required for the patient to conduct activities of daily living in 18 categories, focusing on motor and cognitive function	
		Grading Medical Impairment [145]	Grading rules used for assessing the medical impairment and functional reduction originating from an injury using patients’ medical records before and after the injury	
		Glasgow Outcome Scale [41,81,94,96,125-127,146-149]	A global scale used with patients with brain injuries for rating function outcomes into 5 ordered categories: dead, vegetative state, severe disability, moderate disability, or good recovery	
		Range of motion [89,98,114,137,139,141]	The measurement of movement around a specific joint or body part	
**Biological and physiological outcome indicators**				
	Duration of antibiotic use [34]			The number of days in which individuals are prescribed to receive antibiotic treatment because of injury
	Hematological findings [32,58,70,113]			Laboratory tests on examining the blood content such as hormones, ions, lactates, and inflammatory mediators in the blood
	Cardiovascular findings [32,39,50,81,86,91,92,95,106,113]			Any measures or tests performed related to heart or blood vessels, such as arterial blood pressure, heart rate, and initial heart rhythm
	Histological findings [45]			Results of examination of tissue specimens under a microscope to study the manifestations of a disease
	Renal findings [45,58,150]			Laboratory tests on evaluating kidney function using blood or urine samples, mainly blood urea nitrogen and creatinine
	Toxicological findings [52,116,125,150-152]			Laboratory tests on the type and quantity of substances present in an individual’s body, such as urine toxicology screening
	Metabolic measurements [32,45,70,116,125,152-154]			Measurements related to individuals’ metabolism, such as resting energy expenditure and rewarming speed
	Osteological signs [28,48,49,62,88,104,118,124,137,155,156]			Laboratory tests on the structure and function of bones, such as bone measurements and bone mineral density test
	Neurological signs and findings [41,42,51,52,58,59,63,80,83,86,92,97,104,106,116,142-144,148,157]			A series of tests and measures in examining the function of the brain and the central and autonomic nervous systems, such as intracranial pressure, computed tomography scans, magnetic resonance imaging scans, and cerebral angiography
**Postdischarge outcomes**				
	Long-term behavioral and emotional outcomes [67,135,147,155,158-162]			The long-term outcomes of emotions and behaviors characterized by alteration of feeling tone and by physiological behavioral changes
	Social dependency [163,164]			Capacities necessary for the performance of everyday self-care competence, mobility competence, and social competence
	Possibility of posttraumatic stress disorder or other mental disorders [55,67,97,131,132,135,148,151,159,162,164-166]			The possibility of having mental illnesses that affect one’s mood, thinking, and behavior after experiencing a shocking, scary, or dangerous event
	Presence or absence of disabilities [32,48-50,56,66,74,82,93,101,103,134,135,167]			The presence of disabilities, which refer to impairments, activity limitations, and participation restrictions
	Suicide rate [43,121,159]			The number of people taking their own life after injury per 100,000 population in a period
	Effect on employment or studies [79,106,134,135,140,147,159,161,168]			The consequences of the injury on one’s work life and study life
	Walking distance [28,138]			The distance a person is able to walk in a period, such as the 6-minute walk test
	Quality of life [28,67,70,88,110,111,124,133-136,144,151,162,163,167,169]			The overall enjoyment of life, including aspects of an individual’s sense of well-being, ability to perform various activities, and quality of life with domains of physical functioning, role physical, bodily pain, general health, vitality, social functioning, role emotional, and mental health
	Disability-adjusted life year [67,135,162]			A measure expressed as the number of years lost because of ill health, disability, or early death used to reflect the overall disease burden
	Quality-adjusted life year [67,135,162]			A measure used to reflect the overall disease burden by considering both the quality and quantity of the life lived

^a^ICU: intensive care unit.

^b^AED: accident and emergency department.

^c^DSM-5: Diagnostic and Statistical Manual of Mental Disorders (fifth edition).

### Phase 3 to Phase 4

After review and discussion among the expert panel members, 13 core indicators were identified from the list. The core indicators included the need for operative intervention, infection rate, admission to the intensive care unit (ICU), mortality rate, length of ICU stay, length of hospitalization, AED attendance rate, need for a rehabilitation facility, hospitalization rate after AED, discharge rate, long-term behavioral and emotional outcomes, suicide rate, and disability-adjusted life years (DALYs) per 100,000. Among these 13 indicators, 10 (77%) were from the construct of health care service use and 3 (23%) were from the construct of postdischarge outcomes.

### Phase 5

Table 2 displays the results of the multivariable regression analyses of the associations between core indicators and certified Hong Kong Safe Community status. Applicability tests showed that the Hong Kong Safe Community certification status was not associated with 5 core indicators (admission to ICU, mortality rate, length of ICU stay, need for a rehabilitation facility, and long-term behavioral and emotional outcomes), negatively associated with 4 core indicators (operative intervention, infection rate, length of hospitalization, and DALYs), and positively associated with the remaining 4 core indicators (attendance to AED, discharge rate, suicide rate, and hospitalization rate after attending AED). For example, the Safe Community model implementation was found to reduce the risk of AED attendance (risk ratio=0.65; *P*<.001) and to lower the DALYs per 100,000 (β=−1.91; *P*=.046). These results confirmed the data availability, applicability, and local relevance of the selected core indicators.

**Table 2:** Applicability testing of injury outcome indicators (illustrated by multivariable regression analyses).

Injury outcome indicators	Certified Hong Kong Safe Community	
	Effect (95% CI)^a^	*P* value
Operative intervention, β	−.23 (−0.40 to −0.07)	.007^b^
Infection rates, β (ICD-9CM^c^ 680-686^d^)	−.18 (−0.33 to −0.02)	.03^e^
Admission to ICU^f^, β	.04 (−0.02 to 0.11)	.21
Mortality rate, risk ratio	1.29 (0.98 to 1.69)	.07
Length of stay in ICU, β	−0.02 (−0.08 to 0.04)	.54
Length of hospitalization, β	−1.09 (−1.63 to −0.54)	<.001^g^
Attendance to AED^h^, risk ratio	0.65 (0.64 to 0.65)	<.001^g^
Need for a rehabilitation facility, β (based on discharge destination^i^)	.00 (0.00 to 0.00)	.38
Hospitalization rate after attending AED, β	1.34 (0.47 to 2.22)	.003^b^
Discharge rate, β	.09 (0.04 to 0.15)	.001^b^
Long-term behavioral and emotional outcomes, β (proxy measure: ICD-9CM 905-909^j^)	−0.03 (−0.17 to 0.10)	.64
Suicide rate, risk ratio	1.23 (1.00 to 1.50)	.045^e^
DALYs^k^ per 100,000, β	−1.91 (−3.79 to −0.04)	.046^e^

^a^Adjusted for sex and year of attendance as covariates and district and age groups as random intercepts.

^b^*P*<.01.

^c^ICD-9CM: International Classification of Diseases, Ninth Revision, Clinical Modification.

^d^Infections of skin and subcutaneous tissue.

^e^*P*<.05.

^f^ICU: intensive care unit.

^g^*P*<.001.

^h^AED: accident and emergency department.

^i^Destination to MacMehose Medical Rehabilitation Centre, Cheshire House (Sha Tin, Chun Hom Kok).

^j^Late effects of injuries, poisonings, toxic effects, and other external causes.

^k^DALY: disability-adjusted life year.

## Discussion

### Principal Findings

This study used a multiphased modified Delphi approach to develop a set of core injury outcome indicators specific to the Hong Kong population. These identified indicators have the potential to become standardized tools for the surveillance and evaluation of injury burden and management services in Hong Kong. Specifically, we identified 55 injury outcome indicators from the literature and categorized them into 4 domains: health care service use, functional and psychological outcomes, biological and physiological outcomes, and postdischarge outcomes. On the scoring and ranking by panel experts on data availability, applicability, and validity, 13 indicators were ranked as core indicators because of their high local relevance and reflectiveness of the injury burden in Hong Kong. In addition, we used local hospitalization data to perform applicability testing analyses. These findings support the applicability of these core indicators in local contexts. They would serve as the groundwork for the future establishment of a comprehensive injury surveillance system in Hong Kong, as well as an example of a systematic approach for developing and validating indicators for injury surveillance.

By reviewing the relevant literature, we found that the most common injury outcome construct was health care service use. Approximately 57.7% (82/142) of the reviewed articles used measures of health care service use as indicators of injury outcomes compared with 31.7% (45/142) for functional and psychological outcomes, 21.8% (31/142) for postdischarge outcomes, and 14.8% (21/142) for biological and physiological outcomes. This could be because most measures included in the health care service use construct, such as length of hospitalization, length of stay in ICU, and immediate death status, are frequently used as injury indicators in many countries [54,55,68,69].

It should be noted that other outcome indicator constructs also have their own characteristics. From a clinical perspective, functional and psychological and biological and physiological outcomes can provide information on the holistic impact of an injury on patients. Notably, many functional and psychological outcomes were measured using standardized and psychometrically validated scales or indexes such as the General Health Questionnaire and Glasgow Outcome Scale, which can increase the comparability of results between studies and across countries [170]. Conversely, the biological and physiological construct is difficult to compare, because of its injury-specific and heterogeneous nature. For example, Kraft et al [45] assessed the level of blood hormones in patients with burn injuries, whereas Alanazi et al [153] assessed the level of blood ions in poisoned patients. Owing to these between-study differences in biological outcome measures, we could not compare and determine which injury type may have caused a greater physiological burden on the patient. Thus, it is important to reach a consensus on the most appropriate and readily measurable injury indicators at the biological and physiological level. For instance, some evidence has suggested that injury-associated inflammation is a potentially universal phenomenon among injuries [171-173]. Future research should identify the injury type associated with severe biological and physiological damage and compare inflammatory marker levels (eg, interleukins) between injuries to characterize their respective inflammatory profiles and to examine whether postinjury functional and inflammatory changes would correlate with each other.

Moreover, injury outcome indicators can be time specific. For example, previous studies measured quality of life as an indicator of patients’ perceived outcomes immediately after injury [28,70]. However, considering the fluctuation in the quality of life over time, local experts recommended DALYs and quality-adjusted life years as indicators of outcomes after discharge from the hospital. In addition, there could be overlaps between the domains of injury outcome indicators. More studies are needed to clarify the associations among functional and psychological outcomes, biological and physiological outcomes, and other long-term postinjury outcomes.

From the list of potential indicators, 13 indicators were rated by a panel of experts as suitable for local use. Surprisingly, certification of a Safe Community was associated with higher suicide rates, perhaps as the primary goal of the Safe Community program was to prevent unintentional injuries [25,174], and therefore might be less effective in reducing intentional injuries such as suicidal behaviors. The increase in suicide rate may affect the patterns of estimates of other indicators, as it is often related to more serious consequences and complications [175]. For example, the rate of hospitalization following AED attendance was higher in districts with a Safe Community certification, which could be because of the increased number of suicide cases. In addition, although the estimates of universal injury indicators (eg, length of hospitalization) were reduced in certified Hong Kong Safe Communities, severe injury indicators (eg, mortality rate and ICU admission) showed no differences between districts with and without Safe Community certification. These results suggest that the estimates of severe injury indicators could be influenced by other unmeasured factors than safety measures.

This study developed a set of injury indicators that can be used to evaluate and monitor injury trends and services in Hong Kong. It is evident that a well-established injury surveillance system integrating different data sources can be a valuable tool to assist health care professionals in making better decisions regarding injury trends and preventive services [174,176]. Having demonstrated their functionality and applicability to the context of Hong Kong, health care professionals can use these indicators to develop a better understanding of local injury trends and obtain a more accurate estimate of the impact of injuries on the local health care system. However, challenges exist because of the lack of reliable, sensitive, and standardized data sources for some indicators in Hong Kong [177]. Although the literature review identified indicators in 4 constructs (health care service use, functional and psychological outcomes, biological and physiological outcomes, and postdischarge outcomes), only health care service use and postdischarge outcome indicators were found to have limited applicability as severe injuries are relatively rare in Hong Kong. These findings indicate a lack of postinjury data in local surveillance systems. Moreover, functional and psychological and biological and physiological measures should be integrated as part of routine clinical care for injured patients in Hong Kong. The inclusion of these indicators can help establish a more comprehensive surveillance system to evaluate and monitor injury trends and services more accurately in Hong Kong. Furthermore, assessment methods and tools should be standardized to enhance comparability with other regions.

This study had several limitations. First, gray literature such as government reports was not searched in the review process; hence, we may have missed some relevant indicators, although we consulted experts to confirm whether our list included all important indicators. Second, the Hong Kong Safe Community model was not the most appropriate model for testing the validity of the core injury outcome indicators, as the model cannot address issues related to intentional injuries such as suicide and abuse, which often result in severe consequences and complications. Third, owing to the lack of appropriate data sources in Hong Kong, we could not include all identified injury outcome indicator constructs in the final list of core indicators, which limits the generalizability of the results to other populations.

### Conclusions

This study used a multiphased modified Delphi method to develop a set of indicators to monitor injury trends and burdens in Hong Kong. A total of 55 injury outcome indicators were identified through a literature review and discussed among local experts from different sectors, including the government, health care, community, and academia. A total of 13 indicators were included in the final list of core indicators; however, biological and physiological and functional and psychological outcomes were not included because of the lack of data sources. Model testing results based on a set of core indicator data showed that these core indicators can be applied to Hong Kong settings. The approach used in this study will be a useful example for other cities and regions that aim to systematically tackle the injury burden.

## Abbreviations

AED: accident and emergency department
CDARS: Clinical Data Analysis and Reporting System
DALY: disability-adjusted life year
ICU: intensive care unit

## References

[1] (2010). Injuries and violence: the facts. World Health Organization.

[2] (2014). Action plan to strengthen prevention of unintentional injuries in Hong Kong. Department of Health, Hong Kong Special Administrative Region.

[3] (2010). Injury survey 2008. Surveillance and Epidemiology Branch Centre for Health Protection, Department of Health, Hong Kong Special Adminstrative Region.

[4] Hotz G, Kennedy A, Lutfi K, Cohn S (2009). Preventing pediatric pedestrian injuries. J Trauma.

[5] Theurer WM, Bhavsar AK (2013). Prevention of unintentional childhood injury. Am Fam Physician.

[6] Pike I, Piedt S, Warda L, Yanchar N, Macarthur C, Babul S, Macpherson AK (2010). Developing injury indicators for Canadian children and youth: a modified-Delphi approach. Inj Prev.

[7] Cryer C, Langley J (2007). Developing indicators of injury incidence that can be used to monitor global, regional and local trends. University of Otago.

[8] Cryer C (2003). ICE Injury Indicators Group (ICEIInG) - progress Report, aspirations, goals and strategy development. Proceedings of the International Collaborative Effort on Injury Statistics Volume IV.

[9] Mathers C, Fat DM, Boerma J (2008). The Global Burden of Disease: 2004 Update.

[10] (2017). Hong Kong 2016 Population By-census - Summary Results. Census and Statistics Department, The Government of the Hong Kong Special Administrative Region.

[11] Von Schirnding Y (2002). Health in Sustainable Development Planning: The Role of Indicators.

[12] Pike I, McDonald R, Piedt S, Macpherson A (2014). Developing injury indicators for First Nations and Inuit children and youth in Canada: a modified Delphi approach. Chronic Dis Inj Can.

[13] MacKay M, Vincenten J (2009). Child Safety Report Card 2009: Europe Summary for 24 Countries.

[14] Lyons RA, Brophy S, Pockett R, John G (2005). Purpose, development and use of injury indicators. Int J Inj Contr Saf Promot.

[15] Fink A, Kosecoff J, Chassin M, Brook RH (1984). Consensus methods: characteristics and guidelines for use. Am J Public Health.

[16] Hsu C, Sandford B (2007). The Delphi technique: making sense of consensus. Practical Assess Res Eval.

[17] Arksey H, O'Malley L (2005). Scoping studies: towards a methodological framework. Int J Soc Res Methodol.

[18] Howell D, Molloy S, Wilkinson K, Green E, Orchard K, Wang K, Liberty J (2015). Patient-reported outcomes in routine cancer clinical practice: a scoping review of use, impact on health outcomes, and implementation factors. Ann Oncol.

[19] Archer N, Fevrier-Thomas U, Lokker C, McKibbon KA, Straus SE (2011). Personal health records: a scoping review. J Am Med Inform Assoc.

[20] Rigby MJ, Kohler LI, Blair ME, Metchler R (2003). Child Health Indicators for Europe: a priority for a caring society. Eur J Public Health.

[21] LeMessurier J, O’Donnell S, Walsh P, McRae L, Bancej C (2012). The development of national indicators for the surveillance of osteoporosis in Canada. Chronic Dis Inj Can.

[22] Cryer C, Langley J, Stephenson S (2004). Developing Valid Injury Outcome Indicators: A Report for the New Zealand Injury Prevention Strategy / Colin Cryer, John Langley, Shaun Stephenson.

[23] Canadian Institute for Health Information (2006). Pan-Canadian Primary Health Care Indicators: Pan-Canadian Primary Health Care Indicator Development Project: Report 1, Volume 2.

[24] Harrison JE, Steenkamp M (2002). Technical Review and Documentation of Current NHPA Injury Indicators and Data Sources.

[25] (2017). Hong Kong Safe Community: together we build safe community. Occupational Safety and Health Council.

[26] Lindqvist K, Timpka T, Schelp L, Risto O (2002). Evaluation of a child safety program based on the WHO safe community model. Inj Prev.

[27] Rahimi-Movaghar V (2010). Controlled evaluation of injury in an international safe community: Kashmar, Iran. Public Health.

[28] White T, Guy P, Cooke C, Kennedy S, Droll K, Blachut P, O'Brien PJ (2010). The results of early primary open reduction and internal fixation for treatment of OTA 43.C-type tibial pilon fractures: a cohort study. J Orthop Trauma.

[29] Eroglu O, Sari E, Vural S, Coskun F (2015). Warning: this may be as dangerous as firearm injuries;"grease-gun injury": a case report. Pan Afr Med J.

[30] Almahmoud K, Namas R, Abdul-Malak O, Zaaqoq A, Zamora R, Zuckerbraun B, Sperry J, Peitzman AB, Billiar TR, Vodovotz Y (2015). Impact of injury severity on dynamic inflammation networks following blunt trauma. Shock.

[31] Bagheri SC, Dierks EJ, Kademani D, Holmgren E, Bell RB, Hommer L, Potter BE (2006). Application of a facial injury severity scale in craniomaxillofacial trauma. J Oral Maxillofac Surg.

[32] Toon MH, Maybauer DM, Arceneaux LL, Fraser JF, Meyer W, Runge A, Maybauer MO (2011). Children with burn injuries--assessment of trauma, neglect, violence and abuse. J Inj Violence Res.

[33] Haik J, Liran A, Tessone A, Givon A, Orenstein A, Peleg K, Israeli Trauma Group (2007). Burns in Israel: demographic, etiologic and clinical trends, 1997-2003. Isr Med Assoc J.

[34] Tarim A, Nursal TZ, Basaran O, Yildirim S, Türk E, Moray G, Haberal M (2006). Scalding in Turkish children: comparison of burns caused by hot water and hot milk. Burns.

[35] Cameron PA, Mitra B, Fitzgerald M, Scheinkestel CD, Stripp A, Batey C, Niggemeyer L, Truesdale M, Holman P, Mehra R, Wasiak J, Cleland H (2009). Black Saturday: the immediate impact of the February 2009 bushfires in Victoria, Australia. Med J Aust.

[36] Akçay MN, Oztürk G, Aydinli B, Ozoğul B (2008). Tandir burns: a severe cause of burns in rural Turkey. Burns.

[37] Salem K, Shannak O, Scammell B, Moran C (2014). Predictors and outcomes of treatment in hip hemiarthroplasty dislocation. Ann R Coll Surg Engl.

[38] McClure RJ, Peel N, Kassulke D, Neale R (2002). Appropriate indicators for injury control?. Public Health.

[39] Meghoo CA, Dennis JW, Tuman C, Fang R (2012). Diagnosis and management of evacuated casualties with cervical vascular injuries resulting from combat-related explosive blasts. J Vasc Surg.

[40] Tinkoff G, Esposito T, Reed J, Kilgo P, Fildes J, Pasquale M, Meredith JW (2008). American Association for the Surgery of Trauma Organ Injury Scale I: spleen, liver, and kidney, validation based on the National Trauma Data Bank. J Am Coll Surg.

[41] Jagannathan J, Okonkwo DO, Dumont AS, Ahmed H, Bahari A, Prevedello DM, Jane JA, Jane JA (2007). Outcome following decompressive craniectomy in children with severe traumatic brain injury: a 10-year single-center experience with long-term follow up. J Neurosurg.

[42] Polites S, Sebastian A, Habermann E, Iqbal C, Stuart M, Ishitani M (2014). Youth ice hockey injuries over 16 years at a pediatric trauma center. Pediatrics.

[43] Horrocks J, Price S, House A, Owens D (2003). Self-injury attendances in the accident and emergency department: clinical database study. Br J Psychiatry.

[44] Roberts D, Ouellet J, Sutherland F, Kirkpatrick A, Lall R, Ball C (2013). Severe street and mountain bicycling injuries in adults: a comparison of the incidence, risk factors and injury patterns over 14 years. Can J Surg.

[45] Kraft R, Kulp GA, Herndon DN, Emdad F, Williams FN, Hawkins HK, Leonard KR, Jeschke MG (2011). Is there a difference in clinical outcomes, inflammation, and hypermetabolism between scald and flame burn?. Pediatr Crit Care Med.

[46] Ansari-Moghaddam A, Baghbanian A, Dogoonchi M, Chooban B, Mostaghim-Roudi M, Torkfar G (2013). Epidemiology of burn injuries in south-eastern Iran: a retrospective study. J Pak Med Assoc.

[47] Rimmer RB, Weigand S, Foster KN, Wadsworth MM, Jacober K, Matthews MR, Drachman D, Caruso DM (2008). Scald burns in young children--a review of Arizona burn center pediatric patients and a proposal for prevention in the Hispanic community. J Burn Care Res.

[48] Chua W, De SD, Lin WK, Kagda F, Murphy D (2014). Early versus late flap coverage for open tibial fractures. J Orthop Surg (Hong Kong).

[49] Stannard J, Volgas D, Stewart R, McGwin JG, Alonso J (2009). Negative pressure wound therapy after severe open fractures: a prospective randomized study. J Orthop Trauma.

[50] Yavuz C, Demirtas S, Caliskan A, Ertas F, Kaya H, Aydin M, Benli ED, Celik Y, Eren MN (2013). The predictors of poor outcomes in patients with femoral artery injuries. Eur Rev Med Pharmacol Sci.

[51] Chapman JR, Agel J, Jurkovich GJ, Bellabarba C (2008). Thoracolumbar flexion-distraction injuries: associated morbidity and neurological outcomes. Spine (Phila Pa 1976).

[52] Tsai JR, Sheu CC, Cheng MH, Hung JY, Wang CS, Chong IW, Huang MS, Hwang JJ (2007). Organophosphate poisoning: 10 years of experience in southern Taiwan. Kaohsiung J Med Sci.

[53] Thiels CA, Hernandez MC, Zielinski MD, Aho JM (2016). Injury patterns and outcomes of ice-fishing in the United States. Am J Emerg Med.

[54] Karaca MA, Kartal ND, Erbil B, Öztürk E, Kunt MM, Şahin TT, Özmen MM (2015). Evaluation of gunshot wounds in the emergency department. Ulus Travma Acil Cerrahi Derg.

[55] Pavoni V, Gianesello L, Paparella L, Buoninsegni LT, Barboni E (2010). Outcome predictors and quality of life of severe burn patients admitted to intensive care unit. Scand J Trauma Resusc Emerg Med.

[56] Di Scala C, Gallagher S, Schneps S (1997). Causes and outcomes of pediatric injuries occurring at school. J Sch Health.

[57] Brusselaers N, Hoste EA, Monstrey S, Colpaert KE, De Waele JJ, Vandewoude KH, Blot SI (2005). Outcome and changes over time in survival following severe burns from 1985 to 2004. Intensive Care Med.

[58] Güzel A, Duran L, Paksu S, Akdemir H, Paksu M, Katı C, Başol N, Yılman M, Özsevik SN, Murat N (2013). Drowning and near-drowning: experience of a university hospital in the Black Sea region. Turk J Pediatr.

[59] Zuckerman GB, Gregory PM, Santos-Damiani SM (1998). Predictors of death and neurologic impairment in pediatric submersion injuries. The Pediatric Risk of Mortality Score. Arch Pediatr Adolesc Med.

[60] Biggart MJ, Bohn DJ (1990). Effect of hypothermia and cardiac arrest on outcome of near-drowning accidents in children. J Pediatr.

[61] Con J, Friese RS, Long DM, Zangbar B, O'Keeffe T, Joseph B, Rhee P, Tang AL (2014). Falls from ladders: age matters more than height. J Surg Res.

[62] Backstrom I, MacLennan P, Sawyer J, Creek A, Rue IL, Gilbert S (2012). Pediatric obesity and traumatic lower-extremity long-bone fracture outcomes. J Trauma Acute Care Surg.

[63] Hierholzer C, Bühren V, Woltmann A (2007). Operative timing and management of spinal injuries in multiply injured patients. Eur J Trauma Emerg Surg.

[64] Andruszkow H, Liodakis E, Lefering R, Krettek C, Hildebrand F, Haasper C (2012). Knee injuries in severe trauma patients: a trauma registry study in 3.458 patients. J Trauma Manag Outcomes.

[65] Cardona Cano S, Tiemeier H, Van Hoeken D, Tharner A, Jaddoe VW, Hofman A, Verhulst FC, Hoek HW (2015). Trajectories of picky eating during childhood: a general population study. Int J Eat Disord.

[66] Kraus JF, Fife D, Conroy C (1987). Incidence, severity, and outcomes of brain injuries involving bicycles. Am J Public Health.

[67] Jenewein J, Moergeli H, Wittmann L, Büchi S, Kraemer B, Schnyder U (2009). Development of chronic pain following severe accidental injury. Results of a 3-year follow-up study. J Psychosom Res.

[68] Brusselaers N, Monstrey S, Vogelaers D, Hoste E, Blot S (2010). Severe burn injury in Europe: a systematic review of the incidence, etiology, morbidity, and mortality. Crit Care.

[69] de Carvalho Ponce J, Muñoz DR, Andreuccetti G, de Carvalho DG, Leyton V (2011). Alcohol-related traffic accidents with fatal outcomes in the city of Sao Paulo. Accid Anal Prev.

[70] Eich C, Bräuer A, Timmermann A, Schwarz SK, Russo SG, Neubert K, Graf BM, Aleksic I (2007). Outcome of 12 drowned children with attempted resuscitation on cardiopulmonary bypass: an analysis of variables based on the "Utstein Style for Drowning". Resuscitation.

[71] Schoeneberg C, Kauther M, Hussmann B, Keitel J, Schmitz D, Lendemans S (2013). Gender-specific differences in severely injured patients between 2002 and 2011: data analysis with matched-pair analysis. Crit Care.

[72] Peng J, Wheeler K, Shi J, Groner JI, Haley KJ, Xiang H (2015). Trauma with injury severity score of 75: are these unsurvivable injuries?. PLoS One.

[73] Shinsugi C, Stickley A, Konishi S, Ng CF, Watanabe C (2015). Seasonality of child and adolescent injury mortality in Japan, 2000-2010. Environ Health Prev Med.

[74] Kobusingye O, Guwatudde D, Lett R (2001). Injury patterns in rural and urban Uganda. Inj Prev.

[75] Hill A, Pinto R, Nathens A, Fowler R (2014). Age-related trends in severe injury hospitalization in Canada. J Trauma Acute Care Surg.

[76] Monuteaux M, Lee L, Fleegler E (2012). Children injured by violence in the United States: emergency department utilization, 2000-2008. Acad Emerg Med.

[77] Gofin R, Avitzour M, Haklai Z, Jellin N (2000). Intentional injuries among the young: presentation to emergency rooms, hospitalization, and death in Israel. J Adolesc Health.

[78] Lam NN, Dung NT (2008). First aid and initial management for childhood burns in Vietnam--an appeal for public and continuing medical education. Burns.

[79] Reeb-Whitaker CK, Eckert CM, Anderson NJ, Bonauto DK (2015). Occupational hydrofluoric acid injury from car and truck washing--Washington State, 2001-2013. MMWR Morb Mortal Wkly Rep.

[80] Kim KI, Lee WY, Kim HS, Jeong JH, Ko HH (2014). Extracorporeal membrane oxygenation in near-drowning patients with cardiac or pulmonary failure. Scand J Trauma Resusc Emerg Med.

[81] Nitta M, Kitamura T, Iwami T, Nadkarni VM, Berg RA, Topjian AA, Okamoto Y, Nishiyama C, Nishiuchi T, Hayashi Y, Nishimoto Y, Takasu A (2013). Out-of-hospital cardiac arrest due to drowning among children and adults from the Utstein Osaka Project. Resuscitation.

[82] Centers for Disease Control and Prevention (CDC) (2013). Fatal and nonfatal injuries involving fishing vessel winches--Southern shrimp fleet, United States, 2000-2011. MMWR Morb Mortal Wkly Rep.

[83] Allman FD, Nelson WB, Pacentine GA, McComb G (1986). Outcome following cardiopulmonary resuscitation in severe pediatric near-drowning. Am J Dis Child.

[84] Cummings P, Quan L (1999). Trends in unintentional drowning: the role of alcohol and medical care. JAMA.

[85] Bell N, Cai B (2015). Reliability of the American Community Survey for unintentional drowning and submersion injury surveillance: a comprehensive assessment of 10 socioeconomic indicators derived from the 2006-2013 annual and multi-year data cycles. Inj Epidemiol.

[86] Leroy P, Smismans A, Seute T (2006). Invasive pulmonary and central nervous system aspergillosis after near-drowning of a child: case report and review of the literature. Pediatrics.

[87] Demetriades D, Murray J, Brown C, Velmahos G, Salim A, Alo K, Rhee P (2005). High-level falls: type and severity of injuries and survival outcome according to age. J Trauma.

[88] Gill TM, Murphy TE, Gahbauer EA, Allore HG (2013). The course of disability before and after a serious fall injury. JAMA Intern Med.

[89] Neuman MD, Silber JH, Magaziner JS, Passarella MA, Mehta S, Werner RM (2014). Survival and functional outcomes after hip fracture among nursing home residents. JAMA Intern Med.

[90] Imahara S, Hopper R, Wang J, Rivara F, Klein M (2008). Patterns and outcomes of pediatric facial fractures in the United States: a survey of the National Trauma Data Bank. J Am Coll Surg.

[91] Kaye P, O'Sullivan I (2002). Myocardial contusion: emergency investigation and diagnosis. Emerg Med J.

[92] Gómez PA, de-la-Cruz J, Lora D, Jiménez-Roldán L, Rodríguez-Boto G, Sarabia R, Sahuquillo J, Lastra R, Morera J, Lazo E, Dominguez J, Ibañez J, Brell M, de-la-Lama A, Lobato RD, Lagares A (2014). Validation of a prognostic score for early mortality in severe head injury cases. J Neurosurg.

[93] Perel P, Arango M, Clayton T, Edwards P, Komolafe E, Poccock S, Roberts I, Shakur H, Steyerberg E, Yutthakasemsunt S, MRC CRASH Trial Collaborators (2008). Predicting outcome after traumatic brain injury: practical prognostic models based on large cohort of international patients. BMJ.

[94] Adelson PD, Wisniewski SR, Beca J, Brown SD, Bell M, Muizelaar JP, Okada P, Beers SR, Balasubramani GK, Hirtz D, Paediatric Traumatic Brain Injury Consortium (2013). Comparison of hypothermia and normothermia after severe traumatic brain injury in children (Cool Kids): a phase 3, randomised controlled trial. Lancet Neurol.

[95] Chesnut RM, Marshall LF, Klauber MR, Blunt BA, Baldwin N, Eisenberg HM, Jane JA, Marmarou A, Foulkes MA (1993). The role of secondary brain injury in determining outcome from severe head injury. J Trauma.

[96] Ducrocq SC, Meyer PG, Orliaguet GA, Blanot S, Laurent-Vannier A, Renier D, Carli PA (2006). Epidemiology and early predictive factors of mortality and outcome in children with traumatic severe brain injury: experience of a French pediatric trauma center. Pediatr Crit Care Med.

[97] Ling G, Bandak F, Armonda R, Grant G, Ecklund J (2009). Explosive blast neurotrauma. J Neurotrauma.

[98] Haider AH, Efron DT, Haut ER, DiRusso SM, Sullivan T, Cornwell 3rd EE (2007). Black children experience worse clinical and functional outcomes after traumatic brain injury: an analysis of the National Pediatric Trauma Registry. J Trauma.

[99] Beckman K, Mittendorfer-Rutz E, Lichtenstein P, Larsson H, Almqvist C, Runeson B, Dahlin M (2016). Mental illness and suicide after self-harm among young adults: long-term follow-up of self-harm patients, admitted to hospital care, in a national cohort. Psychol Med.

[100] Cryer C, Langley JD, Jarvis SN, Mackenzie SG, Stephenson SR, Heywood P (2005). Injury outcome indicators: the development of a validation tool. Inj Prev.

[101] Gururaj G (2008). Road traffic deaths, injuries and disabilities in India: current scenario. Natl Med J India.

[102] Smink B, Ruiter B, Lusthof K, de Gier JJ, Uges D, Egberts A (2005). Drug use and the severity of a traffic accident. Accid Anal Prev.

[103] Murdock MA, Waxman K (1991). Helmet use improves outcomes after motorcycle accidents. West J Med.

[104] Schiff MA, Holt VL (2005). Pregnancy outcomes following hospitalization for motor vehicle crashes in Washington State from 1989 to 2001. Am J Epidemiol.

[105] Tefft BC (2013). Impact speed and a pedestrian's risk of severe injury or death. Accid Anal Prev.

[106] Samuelson H, Nekludov M, Levander M (2008). Neuropsychological outcome following near-drowning in ice water: two adult case studies. J Int Neuropsychol Soc.

[107] Finès P, Bougie E, Oliver N, Kohen D (2013). Hospitalizations for unintentional injuries among Canadian adults in areas with a high percentage of Aboriginal-identity residents. Chronic Dis Inj Can.

[108] Randall SM, Fear MW, Wood FM, Rea S, Boyd JH, Duke JM (2015). Long-term musculoskeletal morbidity after adult burn injury: a population-based cohort study. BMJ Open.

[109] Carayanni VJ, Tsati EG, Spyropoulou GC, Antonopoulou FN, Ioannovich JD (2011). Comparing oil based ointment versus standard practice for the treatment of moderate burns in Greece: a trial based cost effectiveness evaluation. BMC Complement Altern Med.

[110] Druery M, Brown TL, La H Brown T, Muller M (2005). Long term functional outcomes and quality of life following severe burn injury. Burns.

[111] Kimmo T, Jyrki V, Sirpa A (1998). Health status after recovery from burn injury. Burns.

[112] Mahendraraj K, Durgan DM, Chamberlain RS (2016). Acute mental disorders and short and long term morbidity in patients with third degree flame burn: a population-based outcome study of 96,451 patients from the Nationwide Inpatient Sample (NIS) database (2001-2011). Burns.

[113] Gregorakos L, Markou N, Psalida V, Kanakaki M, Alexopoulou A, Sotiriou E, Damianos A, Myrianthefs P (2009). Near-drowning: clinical course of lung injury in adults. Lung.

[114] Yaokreh J, Odehouri-Koudou T, Tembely S, Dieth A, Kouamé DB, Ouattara O, Dick K (2012). Delayed treatment of supracondylar elbow fractures in children. Orthop Traumatol Surg Res.

[115] Sekhon LH, Fehlings MG (2001). Epidemiology, demographics, and pathophysiology of acute spinal cord injury. Spine (Phila Pa 1976).

[116] Wang G, Yin S, Shear B, Heard K (2012). Severe poisoning after accidental pediatric ingestion of glycol ethers. Pediatrics.

[117] Olfson M, Gameroff MJ, Marcus SC, Greenberg T, Shaffer D (2005). National trends in hospitalization of youth with intentional self-inflicted injuries. Am J Psychiatry.

[118] El-Khoury F, Cassou B, Charles M, Dargent-Molina P (2013). The effect of fall prevention exercise programmes on fall induced injuries in community dwelling older adults: systematic review and meta-analysis of randomised controlled trials. BMJ.

[119] Lee JC, Tung KT, Li TM, Ho FK, Ip P, Wong WH, Chow C (2017). Fall-related attendance and associated hospitalisation of children and adolescents in Hong Kong: a 12-year retrospective study. BMJ Open.

[120] Sulyman N, Kim MK, Rampa S, Allareddy V, Nalliah RP, Allareddy V (2013). Self Inflicted Injuries among Children in United States - estimates from a nationwide emergency department sample. PLoS One.

[121] Linehan MM, Comtois KA, Brown MZ, Heard HL, Wagner A (2006). Suicide Attempt Self-Injury Interview (SASII): development, reliability, and validity of a scale to assess suicide attempts and intentional self-injury. Psychol Assess.

[122] Tan N, Ang A, Heng D, Chen J, Wong H (2007). Evaluation of playground injuries based on ICD, E codes, international classification of external cause of injury codes (ICECI), and abbreviated injury scale coding systems. Asia Pac J Public Health.

[123] Oliver L, Finès P, Bougie E, Kohen D (2014). Intentional injury hospitalizations in geographical areas with a high percentage of Aboriginal-identity residents, 2004/2005 to 2009/2010. Chronic Dis Inj Can.

[124] Kreder HJ, Rozen N, Borkhoff CM, Laflamme YG, McKee MD, Schemitsch EH, Stephen DJ (2006). Determinants of functional outcome after simple and complex acetabular fractures involving the posterior wall. J Bone Joint Surg Br.

[125] Zakharov S, Navratil T, Salek T, Kurcova I, Pelclova D (2015). Fluctuations in serum ethanol concentration in the treatment of acute methanol poisoning: a prospective study of 21 patients. Biomed Pap Med Fac Univ Palacky Olomouc Czech Repub.

[126] Leach P, Pathmanaban ON, Patel HC, Evans J, Sacho R, Protheroe R, King AT (2009). Outcome after severe head injury: focal surgical lesions do not imply a better Glasgow Outcome Score than diffuse injuries at 3 months. J Trauma Manag Outcomes.

[127] Ewing-Cobbs L, Prasad MR, Kramer L, Cox CS, Baumgartner J, Fletcher S, Mendez D, Barnes M, Zhang X, Swank P (2006). Late intellectual and academic outcomes following traumatic brain injury sustained during early childhood. J Neurosurg.

[128] Kenardy J, Spence S, Macleod A (2006). Screening for posttraumatic stress disorder in children after accidental injury. Pediatrics.

[129] He F, Zhou Q, Zhao Z, Zhang Y, Guan H (2016). Effect of perceived social support and dispositional optimism on the depression of burn patients. J Health Psychol.

[130] McGarry S, Burrows S, Ashoorian T, Pallathil T, Ong K, Edgar DW, Wood F (2016). Mental health and itch in burns patients: potential associations. Burns.

[131] Schäfer I, Barkmann C, Riedesser P, Schulte-Markwort M (2006). Posttraumatic syndromes in children and adolescents after road traffic accidents--a prospective cohort study. Psychopathology.

[132] Wu K, Chan S, Yiu VF (2008). Psychometric properties and confirmatory factor analysis of the posttraumatic stress disorder checklist for Chinese survivors of road traffic accidents. Hong Kong J Psychiatry.

[133] Anzarut A, Chen M, Shankowsky H, Tredget EE (2005). Quality-of-life and outcome predictors following massive burn injury. Plast Reconstr Surg.

[134] Girotto JA, MacKenzie E, Fowler C, Redett R, Robertson B, Manson PN (2001). Long-term physical impairment and functional outcomes after complex facial fractures. Plast Reconstr Surg.

[135] Hours M, Chossegros L, Charnay P, Tardy H, Nhac-Vu H, Boisson D, Luauté J, Laumon B (2013). Outcomes one year after a road accident: results from the ESPARR cohort. Accid Anal Prev.

[136] Whelan D, Litchfield R, Wambolt E, Dainty K, Joint Orthopaedic Initiative for National Trials of the Shoulder (JOINTS) (2014). External rotation immobilization for primary shoulder dislocation: a randomized controlled trial. Clin Orthop Relat Res.

[137] Goldfarb CA, Ricci WM, Tull F, Ray D, Borrelli J (2005). Functional outcome after fracture of both bones of the forearm. J Bone Joint Surg Br.

[138] Musselman K, Fouad K, Misiaszek J, Yang J (2009). Training of walking skills overground and on the treadmill: case series on individuals with incomplete spinal cord injury. Phys Ther.

[139] Angelini F, Helito C, Bonadio M, da Mota E Albuquerque RF, Pecora J, Camanho G (2015). Surgical management of knee dislocations with ligament reconstruction associated with a hinged external fixator. Orthop Traumatol Surg Res.

[140] Manidakis N, Dosani A, Dimitriou R, Stengel D, Matthews S, Giannoudis P (2010). Tibial plateau fractures: functional outcome and incidence of osteoarthritis in 125 cases. Int Orthop.

[141] de Haan J, Schep NW, Zengerink I, van Buijtenen J, Tuinebreijer WE, den Hartog D (2010). Dislocation of the elbow: a retrospective multicentre study of 86 patients. Open Orthop J.

[142] Davey NJ, Smith HC, Wells E, Maskill DW, Savic G, Ellaway PH, Frankel HL (1998). Responses of thenar muscles to transcranial magnetic stimulation of the motor cortex in patients with incomplete spinal cord injury. J Neurol Neurosurg Psychiatry.

[143] Yoon S, Shim Y, Park Y, Chung J, Nam J, Kim M, Park HC, Park SR, Min BH, Kim EY, Choi BH, Park H, Ha Y (2007). Complete spinal cord injury treatment using autologous bone marrow cell transplantation and bone marrow stimulation with granulocyte macrophage-colony stimulating factor: phase I/II clinical trial. Stem Cells.

[144] Casha S, Zygun D, McGowan M, Bains I, Yong V, Hurlbert RJ (2012). Results of a phase II placebo-controlled randomized trial of minocycline in acute spinal cord injury. Brain.

[145] Malm S, Krafft M, Kullgren A, Ydenius A, Tingvall C (2008). Risk of permanent medical impairment (RPMI) in road traffic accidents. Ann Adv Automot Med.

[146] Macpherson P, Teasdale E, Dhaker S, Allerdyce G, Galbraith S (1986). The significance of traumatic haematoma in the region of the basal ganglia. J Neurol Neurosurg Psychiatry.

[147] Olver JH, Ponsford JL, Curran CA (1996). Outcome following traumatic brain injury: a comparison between 2 and 5 years after injury. Brain Inj.

[148] Kazim SF, Shamim MS, Tahir M, Enam SA, Waheed S (2011). Management of penetrating brain injury. J Emerg Trauma Shock.

[149] Berger RP, Beers SR, Richichi R, Wiesman D, Adelson PD (2007). Serum biomarker concentrations and outcome after pediatric traumatic brain injury. J Neurotrauma.

[150] Hedman H, Holmdahl J, Mölne J, Ebefors K, Haraldsson B, Nyström J (2017). Long-term clinical outcome for patients poisoned by the fungal nephrotoxin orellanine. BMC Nephrol.

[151] Zatzick DF, Jurkovich GJ, Gentilello L, Wisner D, Rivara FP (2002). Posttraumatic stress, problem drinking, and functional outcomes after injury. Arch Surg.

[152] Rentschler G, Broberg K, Lundh T, Skerfving S (2012). Long-term lead elimination from plasma and whole blood after poisoning. Int Arch Occup Environ Health.

[153] Alanazi MQ, Al-Jeraisy M, Salam M (2016). Severity scores and their associated factors among orally poisoned toddlers: a cross sectional single poison center study. BMC Pharmacol Toxicol.

[154] Peltz E, D'Alessandro A, Moore E, Chin T, Silliman C, Sauaia A, Hansen KC, Banerjee A (2015). Pathologic metabolism: an exploratory study of the plasma metabolome of critical injury. J Trauma Acute Care Surg.

[155] Su Y, Chen W, Zhang Q, Liu S, Zhang T, Zhang Y (2014). Bony destructive injuries of the calcaneus: long-term results of a minimally invasive procedure followed by early functional exercise: a retrospective study. BMC Surg.

[156] Frey-Rindova P, de Bruin E, Stüssi E, Dambacher M, Dietz V (2000). Bone mineral density in upper and lower extremities during 12 months after spinal cord injury measured by peripheral quantitative computed tomography. Spinal Cord.

[157] Miyanji F, Furlan JC, Aarabi B, Arnold PM, Fehlings MG (2007). Acute cervical traumatic spinal cord injury: MR imaging findings correlated with neurologic outcome--prospective study with 100 consecutive patients. Radiology.

[158] Hammig B, Jozkowski K, Jones C (2014). Injury-related visits and comorbid conditions among homeless persons presenting to emergency departments. Acad Emerg Med.

[159] Sheridan RL, Hinson MI, Liang MH, Nackel AF, Schoenfeld DA, Ryan CM, Mulligan JL, Tompkins RG (2000). Long-term outcome of children surviving massive burns. JAMA.

[160] Wong JS, Brooks D, Inness EL, Mansfield A (2016). The impact of falls on motor and cognitive recovery after discharge from in-patient stroke rehabilitation. J Stroke Cerebrovasc Dis.

[161] Brzuzy S, Corrlgan JD (1996). Predictors of living independently after moderate to severe traumatic brain injury: a comparison study. J Head Trauma Rehabil.

[162] Mayou R, Bryant B (2002). Outcome 3 years after a road traffic accident. Psychol Med.

[163] Peeters GM, Jones M, Byles J, Dobson AJ (2015). Long-term consequences of noninjurious and injurious falls on well-being in older women. J Gerontol A Biol Sci Med Sci.

[164] Ruchholtz S, Pajonk FG, Waydhas C, Lewan U, Nast-Kolb D, Schweiberer L (1999). Long-term results and quality of life after parasuicidal multiple blunt trauma. Crit Care Med.

[165] Bui E, Brunet A, Allenou C, Camassel C, Raynaud J, Claudet I, Fries F, Cahuzac J, Grandjean H, Schmitt L, Birmes P (2010). Peritraumatic reactions and posttraumatic stress symptoms in school-aged children victims of road traffic accident. Gen Hosp Psychiatry.

[166] Logsetty S, Shamlou A, Gawaziuk JP, March J, Doupe M, Chateau D, Hoppensack M, Khan S, Medved M, Leslie WD, Enns MW, Stein MB, Asmundson GJ, Sareen J (2016). Mental health outcomes of burn: a longitudinal population-based study of adults hospitalized for burns. Burns.

[167] Tang D, Li-Tsang CW, Au RK, Li K, Yi X, Liao L, Cao H, Feng Y, Liu C (2015). Functional outcomes of burn patients with or without rehabilitation in Mainland China. Hong Kong J Occup Ther.

[168] Brenneman FD, Redelmeier DA, Boulanger BR, McLellan BA, Culhane JP (1997). Long-term outcomes in blunt trauma: who goes back to work?. J Trauma.

[169] Ly TV, Travison TG, Castillo RC, Bosse MJ, MacKenzie EJ, LEAP Study Group (2008). Ability of lower-extremity injury severity scores to predict functional outcome after limb salvage. J Bone Joint Surg Am.

[170] De Silva MJ, Roberts I, Perel P, Edwards P, Kenward MG, Fernandes J, Shakur H, Patel V, CRASH Trial Collaborators (2009). Patient outcome after traumatic brain injury in high-, middle- and low-income countries: analysis of data on 8927 patients in 46 countries. Int J Epidemiol.

[171] Biffl WL, Moore EE, Moore FA, Peterson VM (1996). Interleukin-6 in the injured patient. Marker of injury or mediator of inflammation?. Ann Surg.

[172] Hur J, Yang HT, Chun W, Kim J, Shin S, Kang HJ, Kim HS (2015). Inflammatory cytokines and their prognostic ability in cases of major burn injury. Ann Lab Med.

[173] Corps KN, Roth TL, McGavern DB (2015). Inflammation and neuroprotection in traumatic brain injury. JAMA Neurol.

[174] Spinks A, Turner C, Nixon J, McClure R (2009). The 'WHO Safe Communities' model for the prevention of injury in whole populations. Cochrane Database Syst Rev.

[175] Plemmons G, Hall M, Doupnik S, Gay J, Brown C, Browning W, Casey R, Freundlich K, Johnson DP, Lind C, Rehm K, Thomas S, Williams D (2018). Hospitalization for suicide ideation or attempt: 2008-2015. Pediatrics.

[176] Chow C, Leung M, Lai A, Chow Y, Chung J, Tong K, Lit A (2012). Development of an electronic emergency department-based geo-information injury surveillance system in Hong Kong. Injury.

[177] Shipton D, Stone DH (2008). The Yorkhill CHIRPP story: a qualitative evaluation of 10 years of injury surveillance at a Scottish children's hospital. Inj Prev.

